# Computational prediction of potential aggravating mechanisms of polyethylene terephthalate microplastics in diabetic foot ulcers: An integrated in silico approach combining network toxicology, bioinformatics, machine learning, and molecular dynamics simulations

**DOI:** 10.1097/MD.0000000000049208

**Published:** 2026-06-05

**Authors:** Dongxiao Li, Zhanhua Ma, Zunwang Li, Zhihong Fu, Hui Guo, Zhaojun Chen

**Affiliations:** a Beijing University of Chinese Medicine Third Affiliated Hospital, Beijing, China; b Beijing University of Chinese Medicine, Beijing, China; c Beijing Miyun District Hospital of Traditional Chinese Medicine (Beijing University of Chinese Medicine Third Affiliated Hospital, Miyun Branch), Beijing, China.

**Keywords:** diabetic foot ulcer, machine learning, molecular docking, polyethylene terephthalate microplastics, toxicology

## Abstract

The increasing incidence of diabetic foot ulcer (DFU) and growing recognition of environmental pollutants have highlighted polyethylene terephthalate microplastics (PET-MP) as a potential metabolic disease trigger. However, the molecular mechanisms linking PET-MP to DFU remain unclear. This study employed integrated network toxicology and bioinformatics to decipher these mechanisms. PET-MP toxicity targets were screened using SwissTargetPrediction and ChEMBL, and DFU-related differentially expressed genes were obtained from GSE199939 and GSE134431. Functional analysis of overlapping genes included gene ontology, Kyoto encyclopedia of genes and genomes, gene set variation analysis, and protein–protein interaction network analysis. Machine learning models (least absolute shrinkage and selection operator, random forest, and support vector machine-recursive feature elimination) and SHapley Additive exPlanations analysis identified key genes, validated via nomogram, molecular dynamics simulation, and molecular docking. From 6723 DFU-related differentially expressed genes, 53 overlapping genes were identified. Functional analysis highlighted pathways including apoptosis, advanced glycation end product–receptor for advanced glycation end-product signaling, arachidonic acid metabolism, and nicotinamide adenine dinucleotide poly-ADP-ribosyltransferase activity. Machine learning and SHapley Additive exPlanations analysis identified PARP10 and PFKFB4 as key genes. Molecular docking revealed moderate binding affinities (Vina scores: ‐6.8 and ‐5.6). Molecular dynamics simulations confirmed conformational stability. PET-MP may exacerbate DFU by disrupting DNA damage repair, enhancing oxidative stress, and impairing glucose metabolism. These in silico findings identify PARP10 and PFKFB4 as potential candidate genes associated with PET-MP-related pathways in DFU, warranting further experimental validation.

## 1. Introduction

In recent years, concerns regarding the risks of polyethylene terephthalate microplastics (PET-MP) have grown significantly within environmental and health circles. This widely used plastic material undergoes physical fragmentation, thermal degradation, and biological processes in natural environments, gradually forming microplastic particles <5 mm in diameter.^[1]^ These particles can be transmitted and accumulated within organisms through the food chain, ultimately entering the human body.^[2]^ While microplastics have been detected in various human organ systems, such as the cardiovascular, dermal, respiratory, and reproductive systems,^[3]^ diabetic foot ulcers (DFU) (a serious diabetic complication primarily driven by hyperglycemia-induced neuropathy) have not been thoroughly examined in the context of this environmental exposure.^[4]^ Hyperglycemia promotes the formation of advanced glycation end products and inflammatory factors, subsequently inducing oxidative stress damage in nerve cells. While poor glycemic control and peripheral neuropathy are established major risk factors for DFU,^[5]^ the role of environmental pollutants like PET-MP remains unclear. Existing research suggests microplastics may cause harm through pathways such as oxidative stress and inflammatory responses,^[6,7]^ yet the molecular mechanisms linking them to DFU progression require further investigation.

Studies on human microplastic exposure indicate a daily dermal contact level of approximately 2.02 MPs/ind/day.^[8]^ Environmental plastic particles can enter the systemic circulation through intestinal absorption and, owing to their hydrophobic nature, also permeate via the skin stratum corneum, sweat glands, hair follicles, and wounds.^[9]^ These multiple routes of entry facilitate the migration and accumulation of microplastics in diabetic foot ulcer wounds. Currently, research on microplastic toxicity has primarily focused on aquatic organisms, while investigations into their impacts on human health remain relatively limited. In vitro experiments have shown^[10]^ that microplastics can induce chromosomal abnormalities, and such genetic damage may be associated with the development and progression of chronic diseases such as diabetes, obesity, and cardiovascular disorders. Moreover, microplastics can exert neurotoxic effects through mechanisms including the activation of oxidative stress pathways and inhibition of acetylcholinesterase activity.^[11,12]^ Animal studies further demonstrate that microplastics in the bloodstream can disrupt hippocampal neuronal membrane structure and impair DNA integrity.^[13,14]^ Notably, neuropathy represents one of the central pathological mechanisms in the onset and progression of diabetic foot ulcers, and the wound healing process itself depends on the stability of DNA and chromosomal structure.

Although preliminary studies have explored the toxic effects of microplastics, in-depth research on the specific pathway interactions between PET-MP and DFU remains limited. Statistics indicate that DFU affects approximately 18.6 million patients globally each year,^[15]^ with its complex pathophysiology involving multiple factors. While existing research has thoroughly examined traditional risk factors such as glycemic control and neuropathy, the potential impact of PET-MP has yet to be systematically evaluated.^[16]^ The molecular basis through which PET-MP exposure influences the pathogenesis of DFU remains poorly characterized.

With the rapid advancement of computational biology and computational chemistry methods, these techniques have demonstrated unique advantages in deciphering complex biological interactions and environmental toxicological mechanisms.^[17]^ They offer novel research avenues for elucidating the toxic effects of microplastics and their molecular interactions with biological systems. Limitations in current evidence highlight the imperative for in-depth investigation into the pathway-specific interactions between PET-MP and DFU. Deciphering these interactions at a molecular level is crucial to unravel the pathophysiological mechanisms of PET-MP in DFU progression. Such insights are pivotal, as they promise to reveal novel therapeutic targets and thereby establish a scientific foundation for public health strategies aimed at mitigating the impact of environmental pollutants on DFU.

This study employs integrated network toxicology and bioinformatics to investigate the mechanisms connecting PET-MP exposure to DFU progression. Specific objectives include: identifying DFU-associated differentially expressed genes under PET-MP exposure and exploring key signaling pathways mediating this process. Our approach involves combining gene expression profiling, functional enrichment analysis, and machine learning to systematically identify toxicological targets and characterize associated pathway-level alterations. Additionally, a nomogram for DFU will be constructed based on key feature genes. Use molecular docking to determine the binding ability of PET-MP with key genes. This study will enhance current understanding of PET-MP’s toxicological effects on DFU and provide scientific rationale for developing targeted strategies to mitigate the adverse impacts of PET-MP exposure on DFU.

## 2. Methods

### 2.1. Data collection and standardization

Gene expression data for DFU were acquired from the gene expression omnibus datasets GSE199939 and GSE134431, encompassing a total of 18 control and 24 DFU samples. Following data acquisition, the Combat algorithm was applied to correct for batch effects. Gene symbol annotations were derived from their respective platform files (GPL24676 for GSE199939 and GPL18573 for GSE134431). The validation set GSE7014 underwent identical standardization to ensure data comparability.

### 2.2. Toxicity target prediction

Recognizing that intact polyethylene terephthalate (PET) microplastic particles lack canonical small-molecule binding pockets, this study employed an exploratory chemical similarity approach to infer potential indirect molecular interference pathways. The Simplified Molecular Input Line Entry System structure of the PET monomeric unit (bis(2-hydroxyethyl) terephthalate analog, CC(=O)C1 = CC = C(C = C1)C(=O)OCCOC) was used as a surrogate probe for the chemical moieties exposed during PET degradation or leaching. Potential interacting proteins were retrieved from SwissTargetPrediction^[18]^ and ChEMBL^[19]^ databases. It is crucial to note that this methodology provides a hypothetical framework for chemical–protein interactions and does not account for the physical stress or particle-induced membrane disruption characteristic of microplastic toxicity. Therefore, the identified targets represent candidate susceptibility factors rather than direct binding receptors for particulate PET.

### 2.3. Differentially expressed gene (DEG) screening

The limma R package (v.3.64.0) was applied to analyze DEGs in DFU samples relative to controls across GSE199939 and GSE134431. Significance thresholds were set at an false discovery rate (FDR)-corrected *P*-value < 0.05 and log fold change > 1. The ComplexHeatmap tool (v.2.24.0) was used to generate a heatmap illustrating the intersection between PET targets and DFU differentially expressed genes.

### 2.4. Functional enrichment analysis

We leveraged the Kyoto encyclopedia of genes and genomes (KEGG) database(https://www.kegg.jp/) to provide comprehensive biological system information. Functional enrichment analysis of the overlapping genes between PET targets and DFU DEGs was then performed for both gene ontology and KEGG pathways using the clusterProfiler R package (v4.16.0). Terms with a significance threshold of FDR-adjusted *P*-value <.05 were visualized using bar and bubble plots.

### 2.5. Gene set variation analysis (GSVA)

Pathway activity differences between the DFU and control groups were assessed using the GSVA R package (v.2.2.0). Reference gene sets were selected from “c5.go.v2025.1.Hs.symbols.gmt” in Molecular Signatures Database. Pathway scores across groups were compared using the limma method, with FDR-corrected *P*-values below .05 considered significant.

### 2.6. Protein–protein interaction (PPI) network analysis

The list of intersecting genes was imported into the STRING database (https://string-db.org/) to construct a PPI network, with a minimum required interaction confidence score set to 0.4. Topological analysis was further performed using Cytoscape (v.3.9.1). we employed its MCODE plugin to identify core functional modules.

### 2.7. Feature gene screening model

We implemented 3 machine learning strategies to screen key biomarkers for DFU. Least absolute shrinkage and selection operator regression analysis was performed using the RLassoCox package (v.1.16.0), employing 10-fold cross-validation to determine the optimal λ value and combining 500 Bootstrap samples to assess feature robustness. The random forest model was constructed using the randomForest package (v.4.7–1.2) with parameters mtry and ntree = 500. Feature importance was assessed based on Gini decay and accuracy decay metrics. The Support Vector Machine model was established using the e1071 package (v.1.7–16). Hyperparameters were optimized through cross-validation and grid search, with Recursive Feature Elimination incorporated to enhance selection efficacy. Finally, a Venn diagram illustrates candidate genes jointly identified by all 3 methods.

### 2.8. SHapley Additive exPlanations (SHAP) analysis

We employed 3 machine learning approaches to construct predictive models for gene expression data associated with microplastic exposure, followed by SHAP analysis using the fastshap package (v.0.1.0) to compute SHAP values for each gene. Through feature importance bar plots and SHAP summary plots (beeswarm plots), we identified key biomarkers and elucidated the complex relationships between gene expression levels and classification predictions.

### 2.9. Prediction model and utility assessment

A regression model was constructed using the rms R package (v.8.0–0) based on the expression levels of key genes. Scores were assigned based on expression differences between DFU and controls for each biomarker, with cumulative scores evaluating individual DFU risk. Model discrimination was assessed via area under the receiver operating characteristic curve (AUC), predictive accuracy via calibration curves, and clinical applicability via decision curve analysis.

### 2.10. Molecular docking

Molecular docking explored PET-target protein binding modes. Protein structures were obtained from the PDB database, and water molecules and irrelevant ligands were removed using PyMOL. The 3D structure of PET was exported from PubChem and converted to the required docking format using AutoDock Tools. A grid was set in the active site of the protein, and AutoDock Vina was used for conformation search and binding energy assessment. The optimal binding mode and its interaction details were ultimately visualized using PyMOL (v.2.5.0).

### 2.11. Molecular dynamics (MD) simulation

MD simulations of the protein–ligand complexes were conducted with the GROMACS 2022.2 package. The systems, set up using the CHARMM36 force field, were solved in a TIP3P water model within a periodic boundary box and neutralized with 150 mM NaCl. After energy minimization and stepwise equilibration in the isothermal–isochoric and isothermal–isobaric ensembles (100 ps each) under positional restraints, a production run was performed for 100 ns using a 2 fs timestep. Trajectories were processed for periodicity and system centering. Subsequent analysis included the root mean square deviation (RMSD) of the protein backbone, radius of gyration, root mean square fluctuation (RMSF), and intermolecular hydrogen bond count.

### 2.12. Ethics statement

This study is purely computational and does not involve any human or animal subjects. All gene expression data were obtained from publicly available databases (gene expression omnibus), which provide de-identified and pre-consented data. Therefore, ethical approval was not required.

## 3. Results

### 3.1. Identification PET-associated toxicity targets in DFU

**Figure 1.**
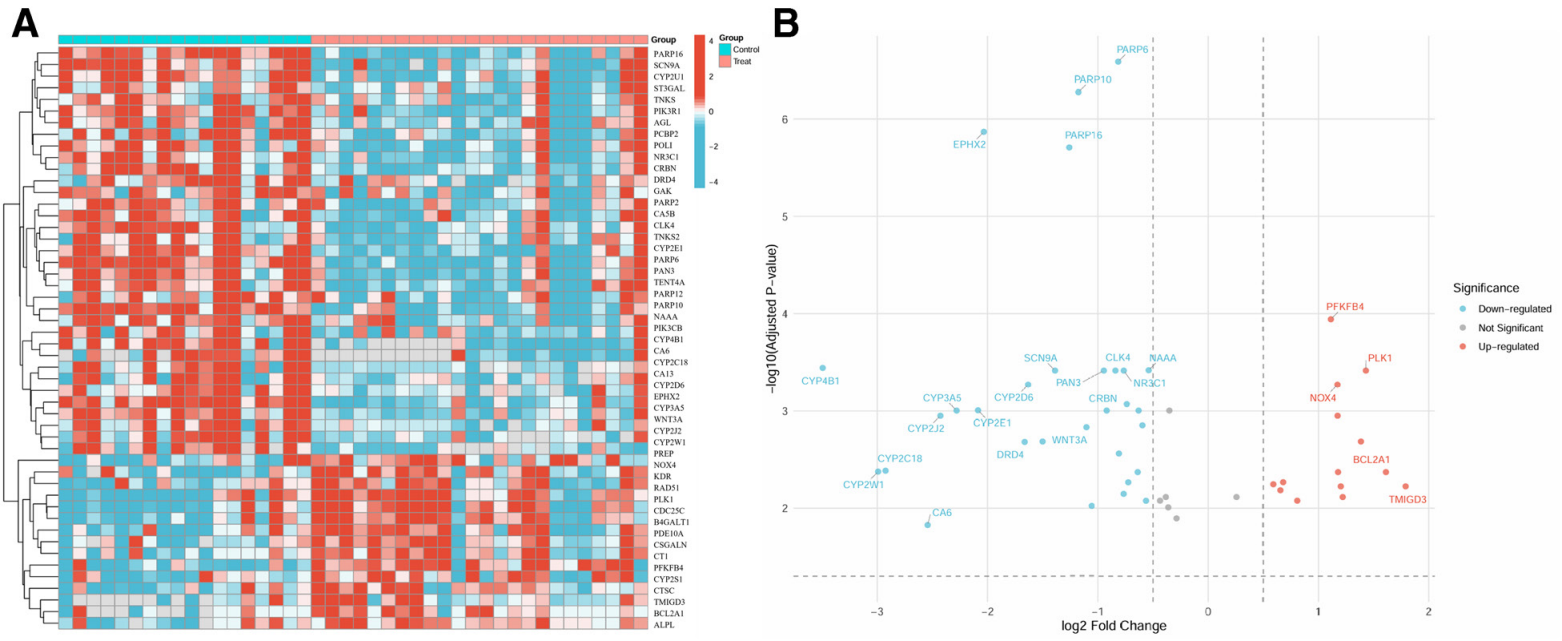
Differential expression patterns of PET-related toxicity targets in diabetic foot ulcers. (A) Heatmap visualization of overlapping gene expression levels for 53 PET-related toxicity targets between GSE199939 and GSE134431 datasets, comparing control and DFU groups. The color scale represents gene expression intensity, with red indicating upregulation and blue indicating downregulation. (B) Volcano plot showing differences in gene expression activity between control and DFU groups. Significantly upregulated genes are marked in red; downregulated genes are marked in blue. PET = polyethylene terephthalate.

Employing the SwissTargetPrediction and ChEMBL databases, we identified 152 potential toxicity targets, which may act as molecular mediators for PET-MP’s toxic effects. Subsequently, analysis of the DFU-related datasets GSE199939 and GSE134431 revealed 6723 DEGs associated with DFU pathogenesis. We found 53 of these DEGs overlapped with PET-associated toxicity targets (representing 0.8% of all DFU-related DEGs). We further visualized these 53 overlapping genes using heatmaps and volcano plots (Fig. 1), plotting their expression profiles across the GSE199939 and GSE134431 datasets. The heatmap clearly revealed differential expression patterns between the control and DFU groups. Comparative analysis revealed significant differential expressions of key genes, highlighting their potential as critical mediators of PET-MP toxicity. These findings substantiate the hypothesis that PET-MP could function as a latent risk factor for DFU by modulating specific genes, thereby establishing a plausible toxicological mechanism for future investigation.

### 3.2. Enrichment analysis

**Figure 2.**
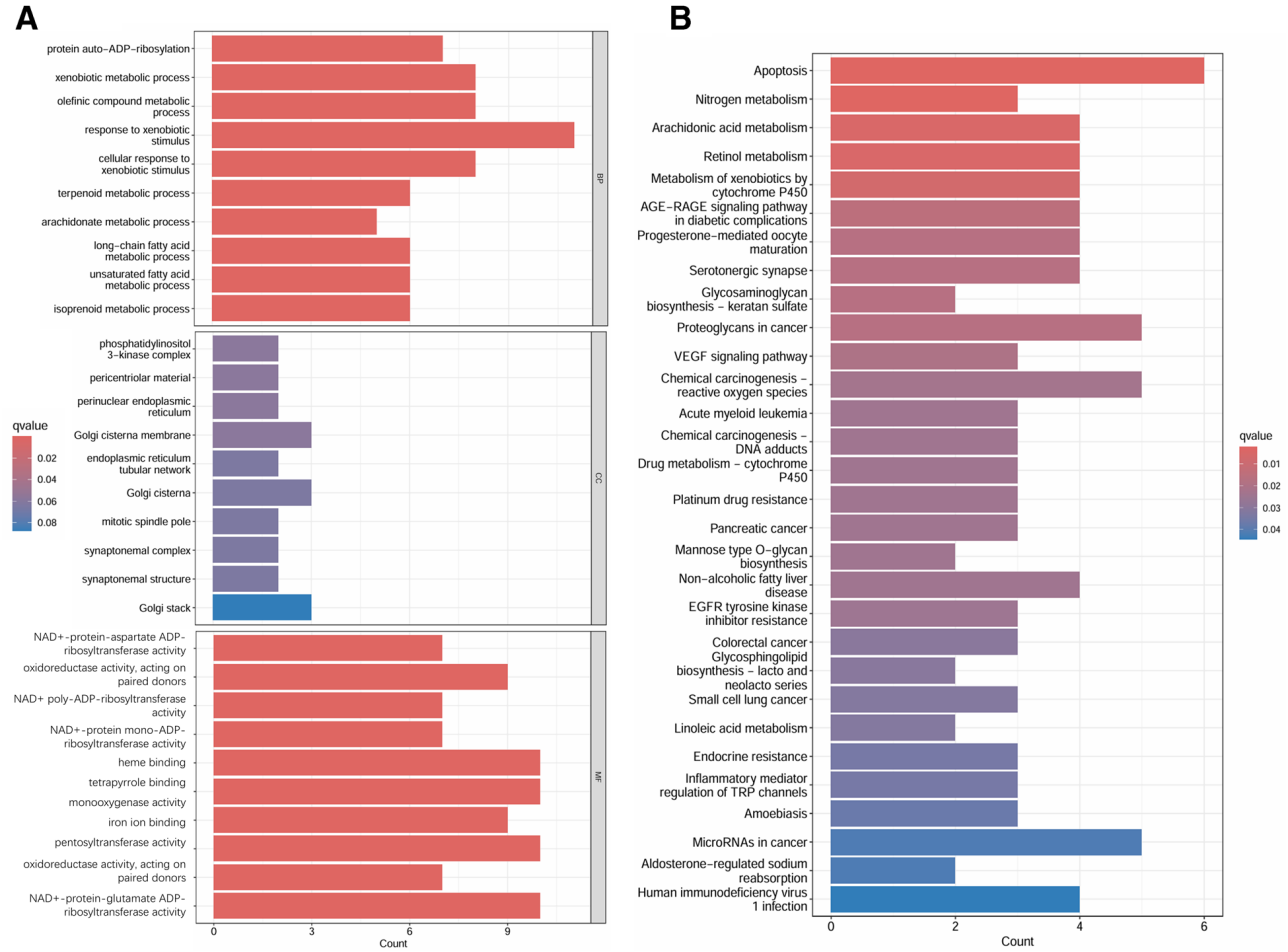
Functional and pathway enrichment analysis of differentially expressed PET toxicity targets. (A) BP enrichment analysis of 53 differentially expressed PET-associated toxicity targets. CC enrichment analysis highlights core cellular structures associated with PET targets. MF enrichment analysis reveals significant terms related to kinase activity and binding. (B) KEGG pathway enrichment analysis for 64 PET-MP toxicity targets. Bar length represents the number of associated genes per pathway; color indicates corrected *P*-value (*q*-value). BP = biological process, CC = cellular component, KEGG = Kyoto encyclopedia of genes and genomes, MF = molecular function, PET-MP = polyethylene terephthalate microplastics.

Enrichment analysis was conducted on the 53 differentially expressed PET-related toxicity targets to investigate the underlying mechanisms of PET-MP in DFU. The biological process (BP) analysis identified significant involvement of the protein auto-ADP-ribosylation pathway, a process closely linked to DNA repair^[20]^ (Fig. 2A). Arachidonic acid metabolism, closely linked to lipid metabolism and inflammatory responses, was also enriched. Cell component enrichment analysis indicated that PET-MP targets were not strongly associated with cellular structures. Conversely, molecular function analysis highlighted significant enrichment in terms related to kinase activity and binding. High enrichment in nicotinamide adenine dinucleotide (NAD^+^) poly-ADP-ribosyltransferase activity underscores that PET-MP may interfere with DNA damage repair, exacerbate oxidative stress, and promote ferroptosis (all factors that could worsen DFU).^[21]^ KEGG pathway enrichment analysis revealed several key pathways potentially affected by PET-MP (Fig. 2B), including apoptosis, the advanced glycation end product–receptor for advanced glycation end-product (AGE–RAGE) signaling pathway, chemical carcinogenesis-reactive oxygen species, and arachidonic acid metabolism. Apoptosis constitutes the direct cytological basis for neurodegeneration and vascular dysfunction.^[22]^ The AGE–RAGE signaling pathway strongly and persistently activates oxidative stress and pro-inflammatory signaling.^[23]^ Pathways including chemical carcinogenesis−reactive oxygen species and arachidonic acid metabolism are known to synergistically promote oxidative stress and inflammation, processes that can culminate in cell necrosis and aggravate DFU.^[24,25]^ This finding underscores the impact of environmental pollutants such as PET-MP on DFU progression.

### 3.3. GSVA analysis

**Figure 3.**
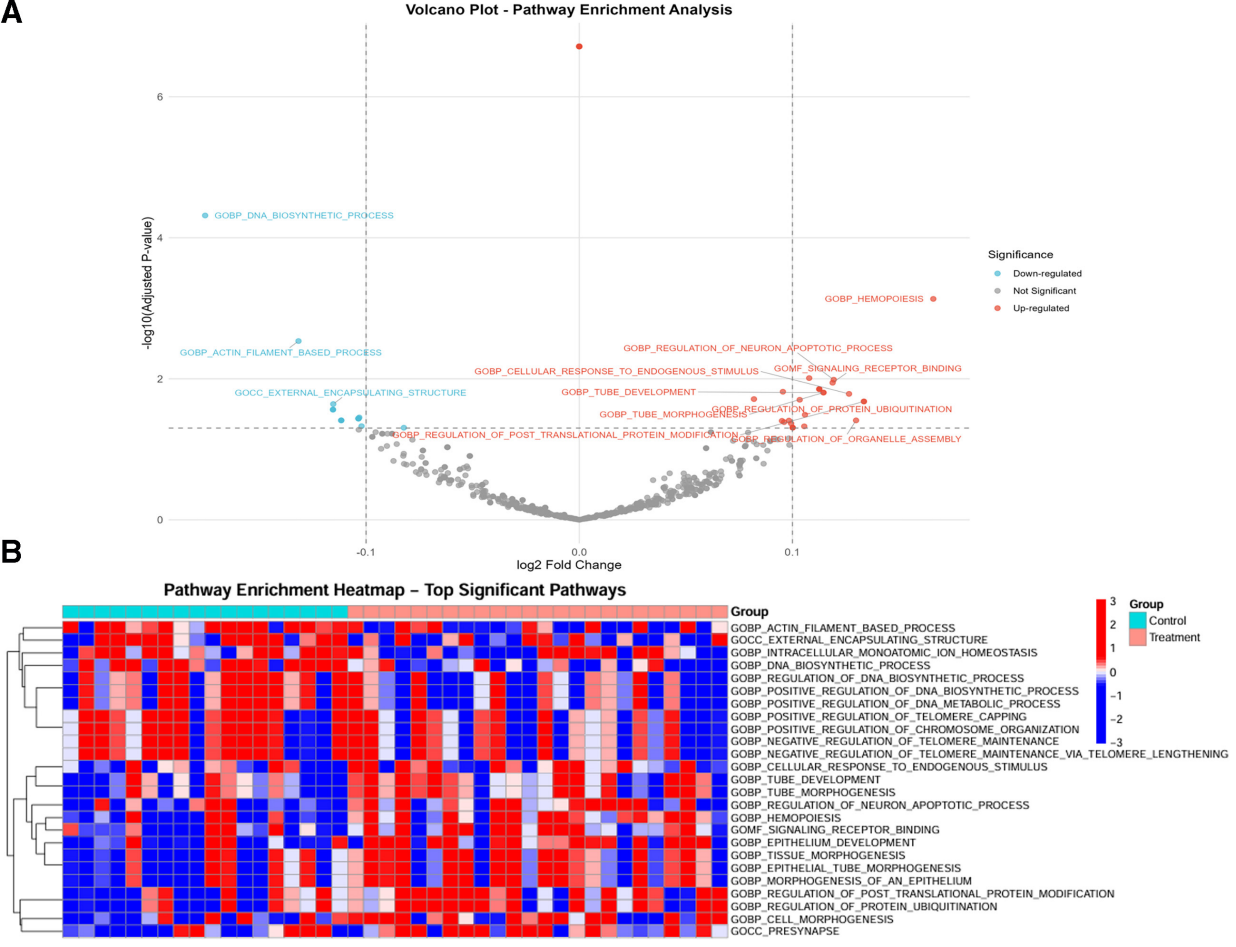
Pathway activity differences between control and diabetic foot ulcer groups. (A) Volcano plot showing pathway activity differences between the control and DFU groups. Significantly upregulated pathways in the DFU group are marked in red, while downregulated pathways are marked in blue. (B) Heatmap visualization of significant pathway activity levels between the CON and DFU groups, selected based on GSVA scores. Each row represents a pathway, with color gradients reflecting pathway activity intensity: red indicates higher activity, blue indicates lower activity. CON = control, DFU = diabetic foot ulcer, GSVA = gene set variation analysis.

We performed GSVA on the 53 PET-related targets to evaluate pathway-level differences between the control and DFU groups. The analysis revealed substantial pathway alterations, with 1334 pathways showing significant dysregulation. As visualized in Figure 3, upregulated pathways are highlighted in red and downregulated in blue. Critically, GSVA indicated the upregulation of 2 key processes: T cell proliferation, which signifies robust adaptive immune activation,^[26]^ and adenylate cyclase activity, a critical step for activating inflammatory cells such as macrophages. Both suggest that PET may induce excessive, uncontrolled inflammatory responses.^[27]^ Downregulated pathways included DNA biosynthesis and regulation-related processes, essentially indicating severely impaired cellular replication and proliferation capabilities.^[28]^ Collectively, these findings converge to suggest that PET-MP exacerbates DFU through a concerted mechanism involving amplified systemic inflammation, dysregulated immune responses, heightened oxidative stress, and impaired DNA biosynthesis. This underscores the pivotal role of environmental pollutants in driving DFU pathogenesis.

### 3.4. PPI network analysis

**Figure 4.**
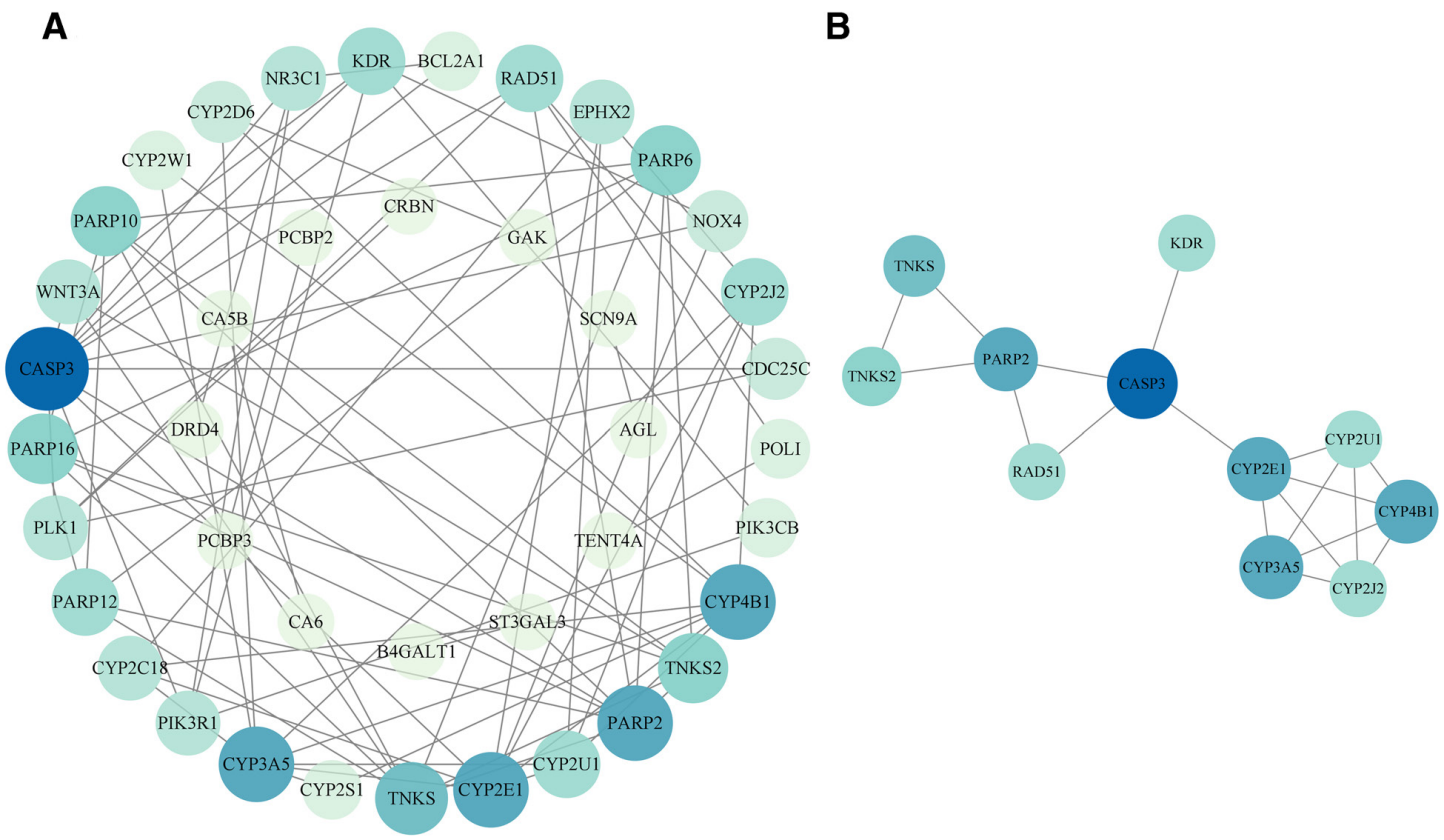
Protein interaction network of PET toxicity targets and key subnetworks. (A) Integrated protein interaction network of 53 differentially expressed PET-related toxicity targets. Nodes represent individual proteins, and edges denote interactions between them, revealing a highly interconnected network structure. (B) Subnetworks highlighting key signaling molecules. PET = polyethylene terephthalate.

To elucidate functional interactions among the 53 PET-related toxicity targets, we constructed a PPI network. The resulting integrated network (Fig. 4A) displays a densely interconnected topology, suggesting high functional interdependence among these proteins. A key submodule was identified (Fig. 4B), containing central signaling molecules such as PARP2, RAD51, TNKS, and TNKS2. These proteins participate in key DNA damage repair pathways, suggesting PET-MP may alter these pathways, leading to genomic instability in DFUs, accelerating cellular senescence or apoptosis, and causing impaired cell proliferation that impedes wound healing.^[29]^ Another prominent cluster includes CASP3, CYP family (CYP2E1, CYP3A5, CYP4B1, CYP2U1, and CYP2J2), which primarily exacerbate oxidative stress and induce apoptosis.^[30]^ These findings underscore the multifaceted potential impact of PET-MPs on DFUs.

### 3.5. Identifying key feature genes using machine learning models

**Figure 5.**
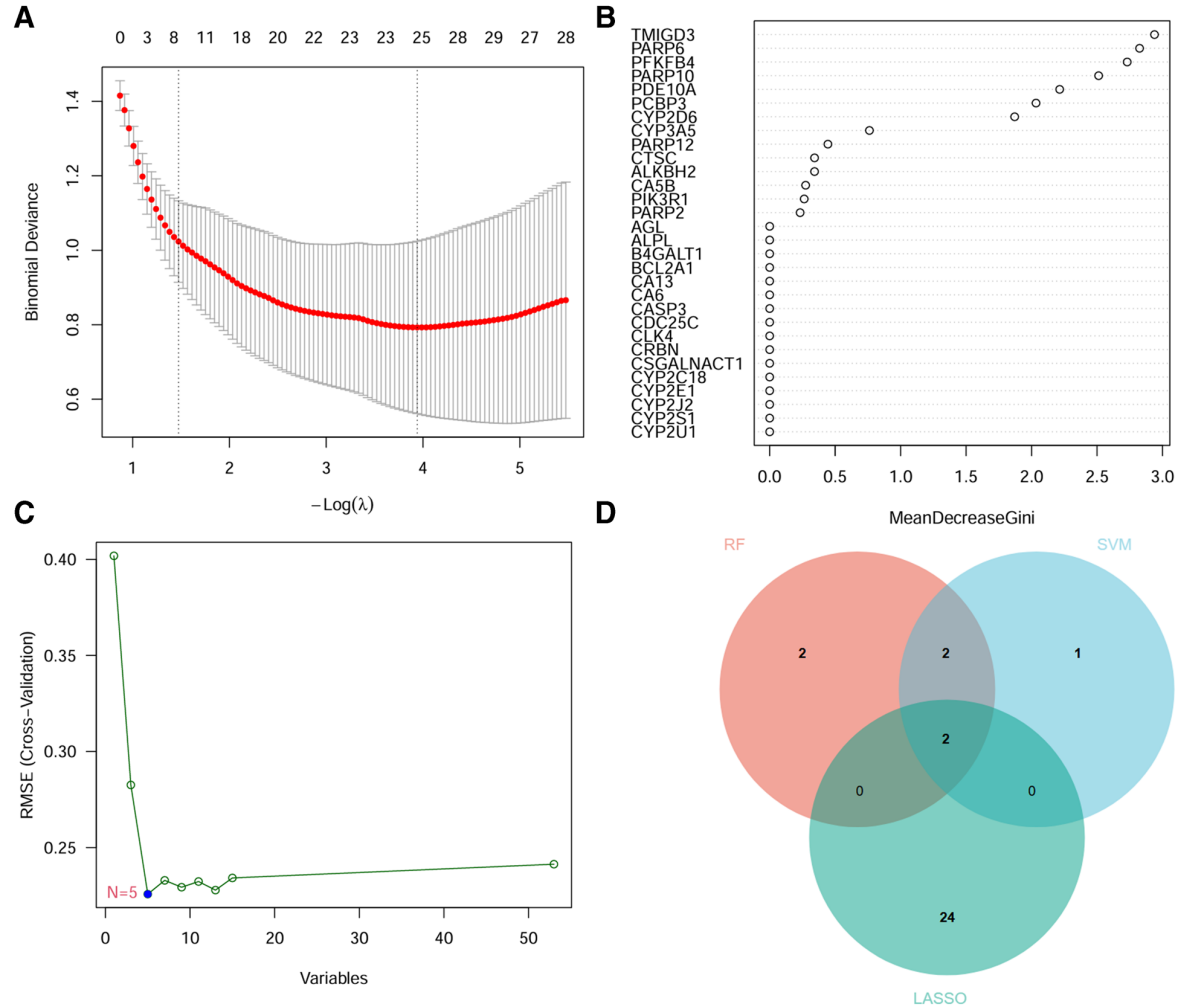
Screening of key feature genes using multiple machine learning algorithms. (A) LASSO regression model coefficient plot for screening significant genes, with optimal λ value selected based on minimum binomial bias. (B) Variable importance ranking in the random forest model based on mean decrease in Gini coefficient. (C) Root mean square error of the SVM-RF model versus the number of variables in the model. The model error is minimal when n = 5 variables, indicating this is the optimal variable subset. (D) Venn diagram summarizing feature gene overlap across the 3 models. LASSO = least absolute shrinkage and selection operator, SVM-RF = Support Vector Machine-Recursive Feature.

We employed 3 machine learning algorithms to identify key feature genes linking PET-MP exposure to DFU. The least absolute shrinkage and selection operator regression model identified features based on the optimal λ value minimizing binomial deviance^[31]^ (Fig. 5A). The random forest model ranked gene importance by the mean decrease in the Gini index^[32]^ (Fig. 5B). The Support Vector Machine-Recursive Feature Elimination model selected an optimal variable subset by minimizing the root mean square error^[33]^ (Fig. 5C). The intersection of feature genes from all 3 models, visualized in a Venn diagram (Fig. 5D), consistently highlighted PARP10 and PFKFB4, underscoring their pivotal role in mediating PET-MP toxicity in DFU.

### 3.6. SHAP analysis

**Figure 6.**
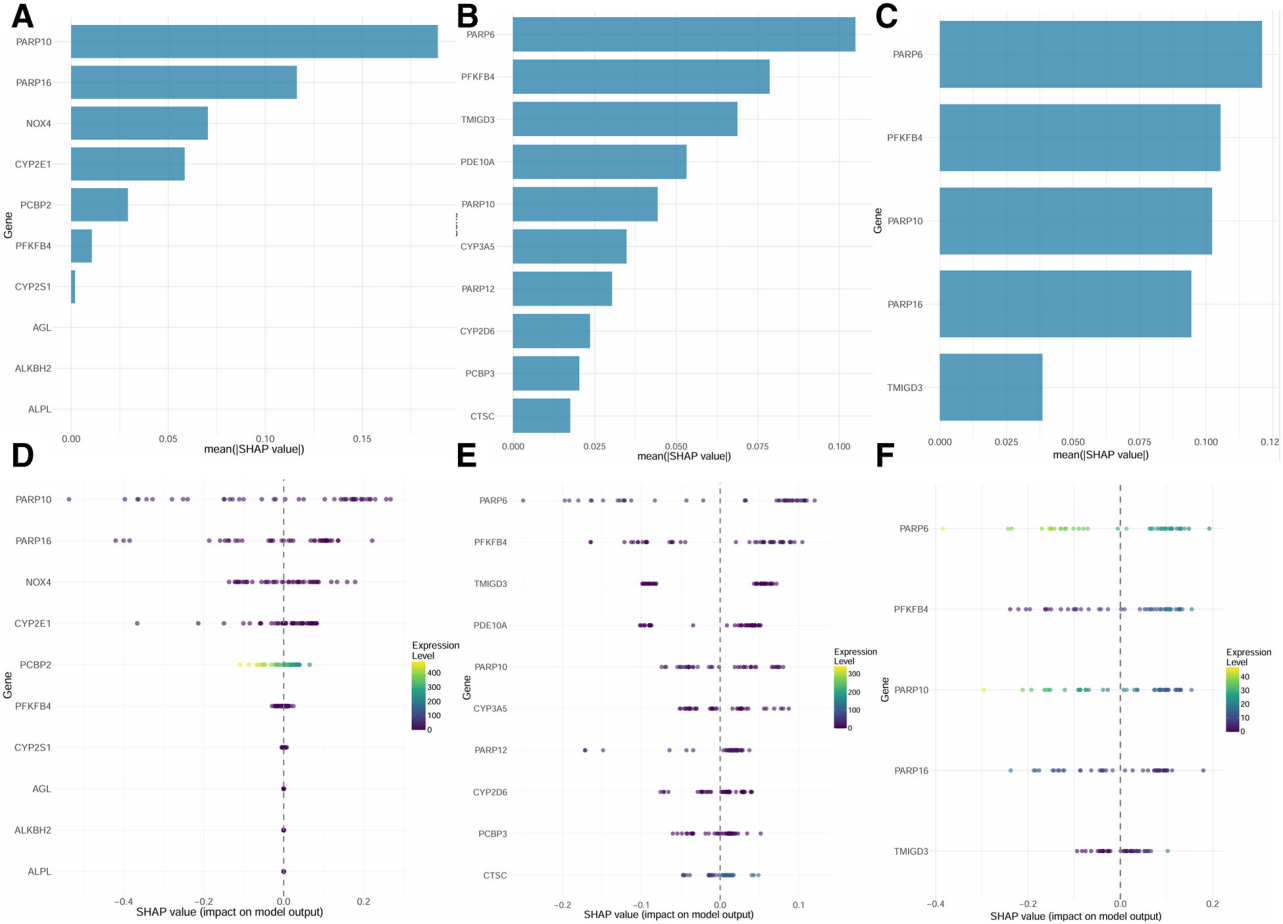
Contribution of Key Feature Genes to Model Prediction (SHAP Analysis). (A–C) SHAP feature importance bar plots for (A) LASSO, (B) random forest, and (C) SVM-RFE models, displaying the top 10 genes ranked by their mean absolute SHAP values. (D–F) Corresponding SHAP summary plots (beeswarm plots) for (D) LASSO, (E) random forest, and (F) SVM-RFE models, where each point represents a single sample, the horizontal position indicates the SHAP value (impact on model output), and color intensity reflects gene expression levels from low (purple) to high (yellow). Positive SHAP values shift predictions toward the microplastic exposure group, while negative values favor the control group classification. LASSO = least absolute shrinkage and selection operator. SHAP = SHapley Additive exPlanations, SVM-RFE = Support Vector Machine-Recursive Feature Elimination.

To elucidate the specific contributions of selected variables to model predictions, we implemented SHAP analysis for comprehensive model interpretation. The SHAP-based feature importance ranking revealed that PARP10 and PFKFB4 consistently demonstrated significant predictive contributions across all computational models (Fig. 6A–C). The SHAP summary plots (Fig. 6D–F) provide sample-level insights, where individual data points represent specific samples and their positions along the *x*-axis reflect SHAP values quantifying each gene’s directional impact on classification outcomes. Positive SHAP values indicate increased probability of assignment to the DFU group, while negative values correspond to higher likelihood of control group classification. The color spectrum represents expression levels, with purple hues denoting reduced gene expression and yellow tones indicating elevated expression patterns.

### 3.7. Construction and validation of a nomogram

**Figure 7.**
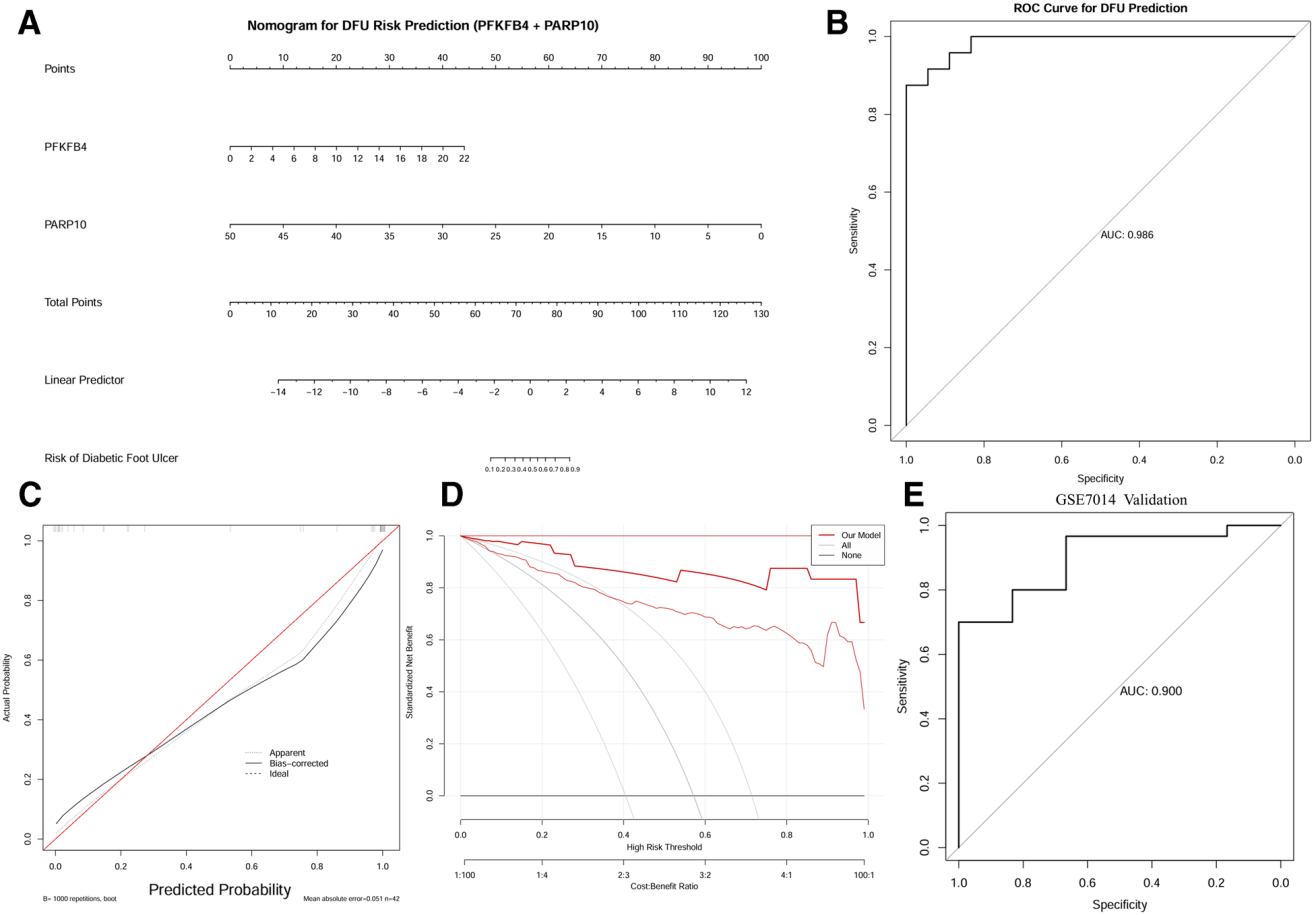
Performance and validation of a DFU risk prediction model based on PFKFB4 and PARP10. (A) Regression plot for diabetic foot ulcer (DFU) risk prediction based on PFKFB4 and PARP10. (B) ROC curve for DFU prediction. The model achieves an area under the curve (AUC) of 0.986 on the training set, indicating excellent discriminative capability. The gray dashed line represents the diagonal line of random guessing. (C) Calibration curve for predicted probabilities. The diagonal line (gray dashed line) represents ideal perfect prediction. The curve (black solid line) and its confidence interval (gray area) show the model’s calibration performance across the entire risk spectrum; closer proximity to the diagonal indicates more accurate predictions. (D) Decision curve analysis. This figure evaluates the clinical net benefit of applying the prediction model at different high-risk thresholds. The model’s net benefit (red solid line) is compared against 2 strategies: all interventions (gray dashed line) and no interventions (black solid line). (E) External validation on the independent validation set GSE7014. The area under the curve (AUC) of 0.900 confirms the model’s strong generalization ability and robustness. ROC = receiver operating characteristic.

To assess the risk of developing DFU in PET-MP-exposed individuals, we constructed a risk prediction score chart using the key genes PARP10 and PFKFB4 identified by the machine learning model. This nomogram (Fig. 7A) provides an intuitive visualization tool for DFU risk by quantifying each gene’s contribution to the risk score.

In the training cohort (GSE199939 and GSE134431 combined), receiver operating characteristic analysis (Fig. 7B) yielded an AUC of 0.986, and the calibration curve (Fig. 7C) demonstrated close agreement between predicted and observed probabilities. However, it is important to acknowledge that performance metrics derived from small-sample, high-dimensional training data may be optimistically biased due to overfitting.

To provide a more rigorous assessment of model generalizability, we evaluated the PARP10/PFKFB4 signature in an entirely independent external validation cohort (GSE7014). As shown in Figure 7E, the model maintained a robust AUC of 0.900, confirming that the discriminatory capacity of the signature extends beyond the training distribution. Decision curve analysis (Fig. 7D) further indicated a positive net benefit across a clinically relevant range of threshold probabilities, suggesting potential utility in risk stratification.

Given the limited sample size of the external cohort (n = 36), we refrain from overinterpreting calibration metrics in that dataset and instead emphasize the consistency of directional association across all 3 independent cohorts. Collectively, these findings support the hypothesis that PET-MP exposure may influence DFU pathogenesis via pathways involving PARP10 and PFKFB4, while underscoring the need for larger prospective studies to validate the clinical applicability of this signature.

### 3.8. Molecular docking

**Figure 8.**
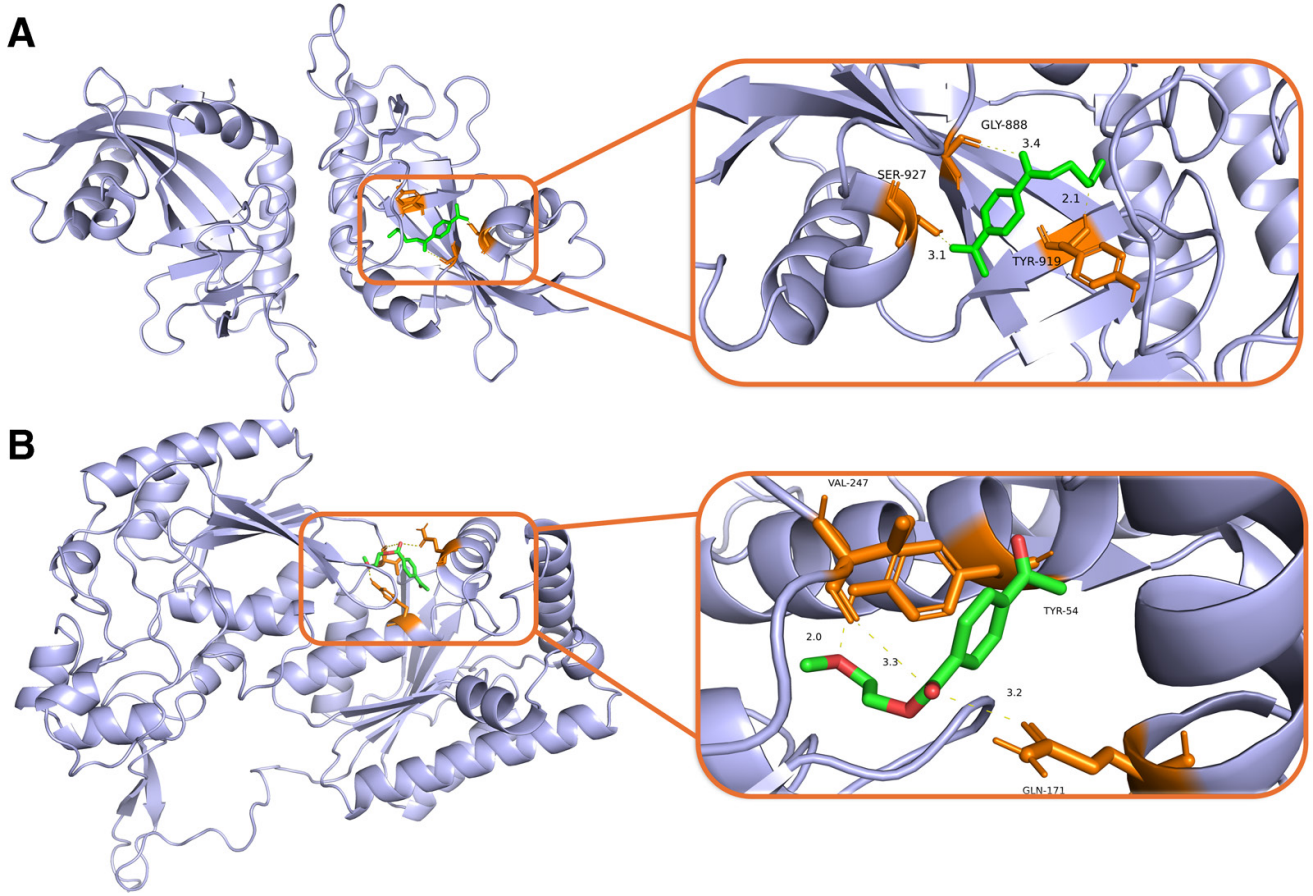
Molecular Docking poses of PET-MP with core target proteins PARP10 and PFKFB4. (A) Molecular interaction view between PET and key residues of PARP10. (B) Interaction view between PET and key residues of PFKFB4. PET = polyethylene terephthalate, PET-MP = polyethylene terephthalate microplastics.

Molecular docking was employed to assess the binding affinity between PET and 2 key DFU-associated proteins. The Vina score for PET–PARP10 was ‐6.8, and that for PET–PFKFB4 was ‐5.6, indicating favorable interactions. The binding of PET to PARP10 (Fig. 8A) revealed interactions with residues GLY-888, SER-927, and TYR919, suggesting potential inhibition of PARP10’s role in DNA damage repair and apoptosis regulation.^[34]^ The PET–PFKFB4 interaction (Fig. 8B) involves residues VAL-247, TYR-54, and GLN-171, indicating possible interference with glucose metabolism reprogramming and oxidative stress.^[35,36]^ The observed binding interactions provide mechanistic support for the hypothesis that PET-MP exacerbate DFU by disrupting key molecular pathways.

### 3.9. MD simulation

MD simulations were further employed to validate the binding affinity of PET-MP with PARP10 and PFKFB4.

**Figure 9.**
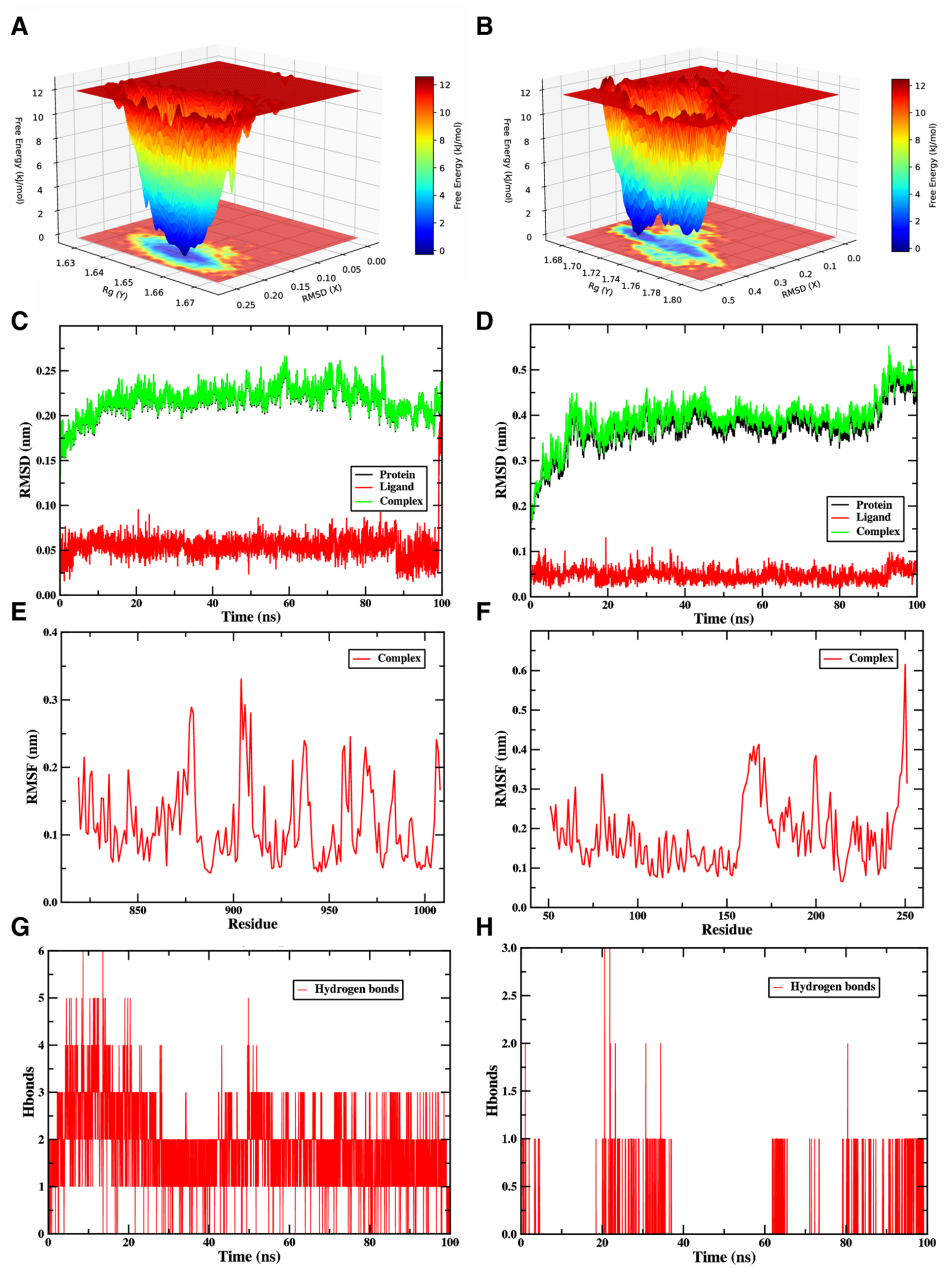
Molecular dynamics simulation analysis of PET-MP complexes with core target proteins. (A and B) Free energy landscapes projected against Rg and RMSD for PET-MP bound to PARP10 (A) and PFKFB4 (B). (C and D) Time evolution of the protein backbone RMSD for the PET-MP–PARP10 (C) and PET-MP–PFKFB4 (D) complexes. (E and F) Residual RMSF profiles for the PET-MP–PARP10 (E) and PET-MP–PFKFB4 (F) complexes. (G and H) Time-dependent count of hydrogen bonds between PET-MP and PARP10 (G) and PFKFB4 (H). PET-MP = polyethylene terephthalate microplastics, Rg = radius of gyration, RMSD = root mean square deviation, RMSF = root mean square fluctuation.

The free energy landscapes (Fig. 9A and B), constructed based on RMSD and radius of gyration, exhibited a single and concentrated low-energy well. This indicates the presence of a well-defined and stable dominant conformation in the PET-MP–PARP10 and PET-MP–PFKFB4 complexes.

RMSD, which reflects the deviation of atomic positions from their initial coordinates, serves as a reliable indicator of conformational stability for both proteins and ligands. Lower RMSD values correspond to higher conformational stability. Both the PET-MP–PARP10 and PET-MP–PFKFB4 systems reached equilibrium after 10 ns (Fig. 9C and D), suggesting that PET-MP binds to PARP10 and PFKFB4 with high stability.

Analysis of the RMSF, which reflects the flexibility of amino acid residues in proteins, revealed relatively low RMSF values (mostly below 0.2 nm) for the PET-MP–PARP10 and PET-MP–PFKFB4 complexes (Fig. 9E and F). This implies reduced flexibility and enhanced structural stability in these systems.

Hydrogen bonds play a crucial role in ligand–protein interactions. Throughout the simulations, the number of hydrogen bonds between PET-MP and the target proteins ranged from 0 to 5. In most frames, the PET-MP–PARP10 complex maintained 2 hydrogen bonds, while the PET-MP–PFKFB4 complex maintained one hydrogen bond (Fig. 9G and H). These results suggest favorable hydrogen bonding interactions between PET-MP and both PARP10 and PFKFB4.

## 4. Discussion

This study offers novel insights into the potential toxicological effects of PET-MP on DFU progression. Through extensive bioinformatics analysis, we propose that PET-MP exposure exacerbates DFU progression by affecting glucose metabolism, oxidative stress regulation, and DNA damage repair. Notably, the identification of PARP10 and PFKFB4 as key signature genes further supports the hypothesis that PET-MP influences DFU through specific molecular interactions, a finding also corroborated by our molecular docking analysis results.

Our research suggests that PET-MP may exacerbate DFU by affecting DNA damage repair. BP enrichment analysis revealed protein auto-ADP-ribosylation as the crucial pathway, a process of protein self-modification. Proteins (typically enzymes) utilize the intracellular coenzyme NAD^+^ as a substrate to covalently attach an ADP-ribose moiety to specific amino acid residues on themselves, a process closely linked to DNA repair.^[20]^ In molecular function enrichment analysis, NAD^+^ poly-ADP-ribosyltransferase activity showed high enrichment, a hallmark activity of core poly (ADP-ribose) polymerase family members (e.g., PARP1).^[21]^ Its primary function is to respond to DNA damage. Upon DNA breaks, PARP1 activates by adding poly-ADP-ribose chains to various nuclear proteins like histones, serving as an “alarm signal.” PET-MP may impair this activity, preventing recruitment of other DNA repair proteins to the damage site. GSVA analysis revealed DNA biosynthesis ranked first among downregulated biological processes. This indicates that PET-MP inhibits cell proliferation and impairs wound healing.^[37]^ A submodule within PPI includes key signaling molecules such as PARP2, RAD51, TNKS, and TNKS2. These proteins participate in critical DNA damage repair pathways, suggesting PET-MP may alter these pathways. This could lead to genomic instability in DFU, accelerate cellular senescence or apoptosis, and cause impaired cell proliferation, ultimately resulting in nonhealing wounds.^[38]^ The key gene PARP10, identified through machine learning as associated with PET-MP exposure, is also closely linked to DNA repair. PARP10 is recruited to DNA damage sites, where it modulates DNA damage response signaling by modifying other proteins via mono-ADP-ribosylation. PET-MP binding to PARP10 disrupts DNA damage repair.^[39]^ Consequently, PET-MP may impair DNA damage repair during the DFU process, thereby exacerbating the condition.

Microplastics can activate oxidative stress in the human body and promote disease progression (a fact we have long been aware of). For instance, recent studies indicate that exposure to various types of microplastics (such as polystyrene) can induce oxidative stress and inflammation, which are major drivers of DFU and other metabolic diseases.^[40]^ We conducted a deeper analysis of the biological processes through which PET-MPs may influence oxidative stress and inflammatory responses in DFUs. BP enrichment analysis revealed significant expressions of arachidonic acid metabolism. This pathway is crucial for cellular responses to external stimuli, suggesting PET-MPs may induce DFUs by disrupting lipid metabolic networks. This disruption generates imbalanced inflammatory and signaling molecules, ultimately leading to cellular dysfunction, inflammation, and oxidative stress through key signaling mechanisms.^[41]^ In KEGG enrichment analysis, apoptosis ranked first, indicating PET-MP induces premature cell death, vascular loss, and neuropathy.^[22]^ Subsequent results showed PET-MP affects the AGE–RAGE signaling pathway, driving oxidative stress, inflammation, and fibrosis, potentially leading to downstream apoptosis.^[23]^ GSVA analysis further revealed enhanced T-cell proliferation and activated adenylate cyclase activity, suggesting robust inflammatory responses induced by PET-MP. The former indicates strong activation of the adaptive immune system with extensive T-cell clonal expansion,^[26]^ while the latter represents a critical step in activating numerous inflammatory cells.^[27]^ This indicates that PET-MP induces excessive, uncontrolled inflammation in diabetic foot wounds. While eliminating pathogens, it also damages normal tissue cells, impeding the repair process. Another significant cluster identified in PPI analysis includes CASP3 and the CYP family (CYP2E1, CYP3A5, CYP4B1, CYP2U1, and CYP2J2), which also exacerbate oxidative stress and induce apoptosis.^[42]^ Thus, PET-MP may exacerbate DFU through pathways such as arachidonic acid metabolism and AGE–RAGE signaling, which intensify oxidative stress and inflammation.

PET-MP may also worsen DFU by affecting glucose metabolism. Repair cells in diabetic foot wounds require substantial energy. Glycolysis driven by PFKFB4 (a key gene associated with PET-MP exposure identified via machine learning) serves as the primary pathway for rapid energy supply.^[35]^ PET-MP may inhibit or disrupt PFKFB4 function, causing cellular “energy depletion” with slowed proliferation and migration. PFKFB4 also suppresses oxidative stress by supporting the pentose phosphate pathway to maintain NADPH and glutathione levels.^[43]^ Thus, PET-MP exacerbates DFU by inhibiting glycolysis via PFKFB4 and disrupting the pentose phosphate pathway pathway to enhance oxidative stress.

While our study provides novel in silico insights into how PET-MP may dysregulate oxidative stress, DNA repair, and glucose metabolism in DFU, several limitations must be acknowledged.First, the findings are derived entirely from computational approaches, including bioinformatics, machine learning, and molecular docking. The moderate binding affinities observed (Vina scores of approximately ‐6.8 and ‐5.6) indicate interactions of intermediate strength, and the use of monomeric Simplified Molecular Input Line Entry System strings as surrogates for microplastic particles represents a necessary reductionist approach given current database constraints. This framework does not account for the physical stress, particle-induced membrane disruption, or surface adsorption phenomena characteristic of microplastic toxicity. Consequently, in vitro and in vivo validation is essential to confirm functional pathway disruption.Second, all gene expression data were sourced from public databases with relatively small sample sizes. Although batch effect correction was applied and an independent external cohort (GSE7014) was used for validation, the moderate sample size of the external dataset (n = 36) limits the precision of calibration assessment and statistical power. The high training AUC (0.986) should be interpreted with caution due to potential overfitting in high-dimensional settings, though the consistent external AUC of 0.900 supports the generalizability of the PARP10/PFKFB4 signature.Third, this study lacks quantitative information on real-world PET-MP exposure levels, tissue bioavailability, and dose-response relationships. The in silico modeled interactions cannot be directly mapped to tissue-level accumulation in DFU wounds, precluding any immediate clinical extrapolation. Fourth, the pathogenesis of DFU is multifactorial, involving hyperglycemia, neuropathy, inflammation, and oxidative stress. PET-MP likely interacts synergistically with these established risk factors, yet the present work does not disentangle these complex interactions.Finally, the long-term and cumulative effects of PET-MP exposure on diabetic wound healing remain uncharacterized. Longitudinal studies and dose–response assessments in relevant animal models are warranted to systematically evaluate the chronic impact of microplastic pollution on DFU progression. In summary, the findings presented herein should be viewed as a hypothesis-generating framework that provides a computational foundation for targeted experimental investigation, rather than definitive mechanistic conclusions.

## 5. Conclusions

This study elucidates the multilevel mechanisms through which PET microplastic exposure may exacerbate DFU pathogenesis, specifically by impairing DNA damage repair, amplifying oxidative stress, and dysregulating glucose metabolism. The identification of core genes including PARP10 and PFKFB4 provides molecular insights into these processes. These findings underscore the pressing need for both increased public health awareness regarding microplastic pollution and further toxicological investigation into their role in DFU. While the current bioinformatics analysis provides substantial evidence, future validation using animal models and clinical specimens will be essential to fully evaluate the human health implications of PET microplastic exposure.

## Acknowledgments

The authors thank the Beijing Miyun District Health Commission for financial support through the Miyun District Traditional Chinese Medicine New Era 125 Project (New 125 Talents). The funder had no role in study design, data collection, analysis, decision to publish, or preparation of the manuscript. The authors also thank the public databases (GEO, SwissTargetPrediction, ChEMBL, etc) for providing accessible data. The authors declare that they have no known competing financial interests or personal relationships that could have appeared to influence the work reported in this paper.

## Author contributions

**Conceptualization:** Dongxiao Li, Zhanhua Ma.

**Data curation:** Zunwang Li, Zhihong Fu.

**Formal analysis:** Hui Guo.

**Funding acquisition:** Zhaojun Chen.

**Software:** Dongxiao Li.

**Writing – original draft:** Dongxiao Li, Zhanhua Ma.

**Writing – review & editing:** Zhaojun Chen.
